# FLRT3 and TGF‐β/SMAD4 signalling: Impacts on apoptosis, autophagy and ion channels in supraventricular tachycardia

**DOI:** 10.1111/jcmm.18237

**Published:** 2024-03-20

**Authors:** Yang Pang, Ye Xu, Qingxing Chen, Kuan Cheng, Yunlong Ling, Jun Jang, Junbo Ge, Wenqing Zhu

**Affiliations:** ^1^ Department of Cardiology, Shanghai Institute of Cardiovascular Diseases, Zhongshan Hospital Fudan University Shanghai China; ^2^ State Key Laboratory of Genetic Engineering, Institute of Genetics, School of Life Science Fudan University Shanghai China

**Keywords:** FLRT3, Na^+^ and K^+^ channel, supraventricular tachycardia, TGF‐β/SMAD4 singling pathway

## Abstract

To explore the underlying molecular mechanisms of supraventricular tachycardia (SVT), this study aimed to analyse the complex relationship between FLRT3 and TGF‐β/SMAD4 signalling pathway, which affects Na^+^ and K^+^ channels in cardiomyocytes. Bioinformatics analysis was performed on 85 SVT samples and 15 healthy controls to screen overlapping genes from the key module and differentially expressed genes (DEGs). Expression profiling of overlapping genes, coupled with Receiver Operating Characteristic (ROC) curve analyses, identified FLRT3 as a hub gene. In vitro studies utilizing Ang II‐stimulated H9C2 cardiomyocytes were undertaken to elucidate the consequences of FLRT3 silencing on cardiomyocyte apoptosis and autophagic processes. Utilizing a combination of techniques such as quantitative reverse‐transcription polymerase chain reaction (qRT‐PCR), western blotting (WB), flow cytometry, dual‐luciferase reporter assays and chromatin immunoprecipitation polymerase chain reaction (ChIP‐PCR) assays were conducted to decipher the intricate interactions between FLRT3, the TGF‐β/SMAD4 signalling cascade and ion channel gene expression. Six genes (AADAC, DSC3, FLRT3, SYT4, PRR9 and SERTM1) demonstrated reduced expression in SVT samples, each possessing significant clinical diagnostic potential. In H9C2 cardiomyocytes, FLRT3 silencing mitigated Ang II‐induced apoptosis and modulated autophagy. With increasing TGF‐β concentration, there was a dose‐responsive decline in FLRT3 and SCN5A expression, while both KCNIP2 and KCND2 expressions were augmented. Moreover, a direct interaction between FLRT3 and SMAD4 was observed, and inhibition of SMAD4 expression resulted in increased FLRT3 expression. Our results demonstrated that the TGF‐β/SMAD4 signalling pathway plays a critical role by regulating FLRT3 expression, with potential implications for ion channel function in SVT.

## 1 INTRODUCTION

Supraventricular tachycardia (SVT) is a type of cardiac arrhythmia defined as an abnormally fast and regular heart rhythm that emanates from the atrium region of the heart located above the ventricles. There are many subtypes of SVT, clinically mainly called atrioventricular nodal reentrant tachycardia (AVNRT) and atrioventricular reciprocating tachycardia (AVRT), which are caused by the reentrant mechanism.^1^ While various factors such as age, exercise and genetics contribute to the onset of SVT, the precise pathogenesis remains elusive.^2^, ^3^ Previous studies had reported aberrations in ion channel activity within cardiomyocytes, alterations in cellular communication, and dysregulated calcium handling can be intricately linked with SVT pathophysiology.^4^, ^5^ Despite the relief offered by current therapeutic interventions like drug treatments and catheter ablation,^6^, ^7^ some cases persistently resist treatment or are prone to recurrence. This accentuates an urgent need for innovative diagnostic tools, treatments and prognostic markers at the myocardial cellular level to optimize the management of SVT and enhance patient outcomes.

The primary regulators of cardiac action potentials and contributors to reentrant formation are sodium and potassium ion channel proteins. In SVT, alterations in the expression, function or regulation of these ion channel proteins can disrupt normal cardiac electrical conduction, leading to tachyarrhythmias.^8^, ^9^ Alterations in these ion channels, pivotal for maintaining cardiac action potentials, can skew the delicate balance of electrical conduction in the heart.^10^ The latest investigation has unveiled the pivotal involvement of cancer‐associated fibroblasts (CAFs) in the heightened aggressiveness of colorectal cancer. This phenomenon is attributed to the effective downregulation of FLRT3 expression facilitated by the activation of the TGF‐β/SMAD4 signalling pathway.^11^ In addition, another study demonstrated that targeting SMAD4 in adult cardiomyocytes resulted in the deletion of classical TGF‐β signalling, which triggered the downregulation of several ion channel genes.^12^ The study also revealed that the N‐terminal region of Smad4 plays an integral role in mediating the inhibition of trans‐epithelial sodium transport, further supporting the possibility that TGF‐β1 reduces epithelial sodium channel function through a Smad4‐dependent pathway. Andreasen L performed next‐generation sequencing on 67 genes in 298 AVNRT patients and 10 family members using the HaloPlex Target Enrichment System.^13^ They discovered 229 gene variants across 60 genes. Notably, 75 of the 284 AVNRT patients and three related family members exhibited variants influencing sodium handling. Meanwhile, 54 of these 284 patients showed variations impacting the heart's calcium management. This suggests that AVNRT could be an electrical arrhythmic disorder linked to irregular sodium and calcium management. Based on these findings, this study for the first time explored a number of AVNRT‐related genes through gene sequencing on collected patients, which is of great significance for revealing the pathological mechanism of AVNRT. Myocardial fibrosis was another factor that may influnce the regional myocardial conduction velocity and the formation of an reentrant. Cell autophagy and apoptosis are fundamental cellular processes that regulate cell survival and may attribute to the process of myocardial fibrosis.^14^, ^15^ Recent evidence suggests that apoptosis contributes to the progression of cardiac hypertrophy, a maladaptive enlargement of heart muscle cells.^16^ Moreover, autophagy plays a crucial role in cardiovascular diseases, including cardiac hypertrophy, and modulating autophagic activity can inhibit cardiac hypertrophic responses.^17^, ^18^ Drawing from the above, it becomes imperative to delve deeper into the nuanced interplay between ion channels, autophagy, apoptosis and signalling pathways in SVT patients. Such explorations stand to enrich our understanding and open avenues for more precise therapeutic interventions.

Given the intricate involvement of cardiomyocytes in SVT pathogenesis and the interdependent roles of ion channels, autophagy and apoptosis in cardiovascular conditions, a comprehensive understanding of the molecular interplay is necessary. By successfully determining the optimal soft‐threshold power for co‐expression network construction and identifying key modules relevant to SVT, we established a powerful framework for further research. Differentially expressed genes (DEGs) and their associated functional analysis revealed potential molecular players and pathways involved in SVT pathogenesis, revealing underlying mechanisms. Furthermore, the discovery of overlapping genes bridging DEGs and co‐expression modules highlighted a critical intersection worthy of further exploration. This research not only contributes to our understanding of SVT but also paves the way for the development of diagnostic markers and treatment strategies that will ultimately benefit individuals affected by this disease and improve patient outcomes.

## 2 MATERIALS AND METHODS

### 2.1 Patient information

There were 15 controls and 85 SVT patients (46 AVNRT patients diagnosed in electrophysiological studies and 39 AVRT patients) recruited from Shanghai Zhongshan Hospital for this study from 2019 to 2020 for the sequencing analysis. Blood samples were collected from all participants under sinus rhythm. Patients with structural heart disease were removed from the study. All the patients in this study reported no family history of SVT. The study got the permission of the ethics committee of Zhongshan Hospital, Fudan University (B2018‐065). Refer to the attached Table S1 for detailed patient information.

### 2.2 Construction of weighted gene co‐expression network

Based on the gene expression from our selected samples (15 controls and 85 SVT patients), we conducted a weighted gene co‐expression network analysis (WGCNA). Using the R package WGCNA (version 4.0.1), we constructed a co‐expression network incorporating all genes, applying a soft threshold of 9. To evaluate internal network connection, the weighted adjacency matrix was initially converted into a topological overlap matrix (TOM). Hierarchical clustering was then employed to generate a dendrogram showing the hierarchical structure of the TOM matrix. In this dendrogram, branches symbolize distinct gene modules, each denoted by a unique colour. Genes were organized into modules derived from their expression patterns and weighted correlation coefficients, leading to the categorization of numerous genes into distinct modules. The module emphasized in blue demonstrated the most substantial correlation with our samples, exhibiting a coefficient of 0.107. Consequently, it was selected for in‐depth analysis in our study.

### 2.3 Identification and enrichment analysis of DEGs

To identify DEGs between the 15 controls and 85 SVT patients, we utilized the ‘limma’ package in R (version 4.1.0). Genes with a fold change (FC) greater than 1 were considered upregulated, while those with FC less than 1 were considered downregulated. Both criteria required a statistical significance threshold of *p* < 0.001. Visualization of the differential expression was achieved using the ‘pheatmap’ package in R. Subsequently, to gain deeper insights into the biological functions and pathways associated with the DEGs, a comprehensive functional enrichment analysis was conducted using the Enrichr database (https://maayanlab.cloud/Enrichr/). This included Gene Ontology (GO) terms, covering Biological Processes (BP), Cellular Components (CC) and Molecular Functions (MF), as well as pathways from the Kyoto Encyclopedia of Genes and Genomes (KEGG). Results with a *p*‐value below 0.05 were deemed statistically significant.

### 2.4 Analysis of expression and diagnostic capabilities of overlapping genes

We employed the ‘VennDiagram’ package in R (version 1.6.20) to analyse the overlapping genes between the blue module and the DEGs. After this, we examined the expression patterns of these overlapping genes in both control and case samples. To evaluate the clinical diagnostic potential of the overlapping genes, we utilized the time‐dependent receiver operating characteristic (tROC) approach, calculating the area under the curve (AUC) for each gene.

### 2.5 Acquisition of data sets and expression analysis of the hub gene

The GSE79768 data set and the GSE115574 data set were obtained from the Gene Expression Omnibus (GEO; https://www.ncbi.nlm.nih.gov/geo/) database. Among them, the GSE79768 data set includes 14 atrial fibrillation (AF) samples (as the case group) and 12 sinus rhythm (SR) samples (as the control group). The GSE115574 data set includes 15 permanent AF samples (as the case group) and 31 SR samples (as the control group). Then, we analysed the expression of the hub gene in this study in different samples of the two data sets through the Wilcoxon rank‐sum test, and performed visual analysis through the ‘ggplot2’ package of R software.

### 2.6 JASPAR database

The JASPAR (http://jaspar.genereg.net/) database provides a comprehensive collection of transcription factor binding profiles and matrices.^19^ It provides valuable insights into potential binding motifs that transcription factors may recognize within gene promoter regions.^20^ In this study, the JASPAR database was used to predict the binding sites of FLRT3 and SMAD4.

### 2.7 Cell culture and treatment

H9C2 cardiomyocytes, originating from rat cardiac tissue, were sourced from ATCC. These cells were cultured in DMEM enriched with 10% FBS and 1% penicillin–streptomycin, kept in a 37°C incubator with a 5% CO_2_ atmosphere and proper humidity. For the induction of cardiomyocyte hypertrophy, cells were exposed to 9 μM angiotensin II (Ang II) for 24 h. In addition to the angiotensin II treatment, the cells were also treated with varying concentrations of TGF‐β, specifically 0.1, 1, 2 and 5 ng/mL, for two different durations: 30 min and 48 h.

### 2.8 Cell transfection

For transient transfection, H9C2 cells were seeded in 24‐well plates at a density of 2 × 10^5^ cells per well. To achieve knockdown of FLRT3 expression, cells were transfected with two specific small interfering RNAs (siRNAs), namely si‐FLRT3‐1 and si‐FLRT3‐2. Additionally, SMAD4 expression was also targeted for knockdown using two distinct siRNAs (si‐SMAD4‐1 and si‐SMAD4‐2). Transfection was carried out using Lipofectamine 3000 (Invitrogen, USA), following the manufacturer's recommendations. Post‐transfection, cells were incubated for an optimal duration to ensure effective downregulation of FLRT3 and SMAD4 expressions. To monitor the endogenous expression of FLRT3, the cells were infected with a lentivirus encoding Flag‐FLRT3. After viral infection, cells were allowed to recover for 48 h before being analysed.

### 2.9 Quantitative reverse‐transcription polymerase chain reaction (qRT‐PCR)

RNA from H9C2 cells was isolated using TRIzol (Thermo Fisher Scientific, USA) as per the recommended guidelines. The PrimeScript RT Reagent Kit (Takara, Japan) facilitated cDNA generation. qRT‐PCR, utilizing SYBR Green PCR Master Mix (Applied Biosystems, USA), was conducted on a StepOnePlus Real‐Time PCR platform (Applied Biosystems, USA). Gene expression was referenced against GAPDH and assessed through the 2^−ΔΔCT^ technique. The specific primer sequences utilized in this study for FLRT3, SCN5A, ANP, BNP, KCNIP2, KCND2, SMAD4 and β‐actin are provided in Table 1.

**TABLE 1:** Primer sequences for qRT‐PCR.

Genes	Sequences
FLRT3	F: 5′‐GGAGGAGAAGAAAGGATGACTATG‐3′
	R: 5′‐CGAGATGGGTTCATTGCTTATTG‐3′
SCN5A	F: 5′‐TGCATCTGGGAAATCTAGGC‐3′
	R: 5′‐GCAAGTTCCTCCAAACAGGA‐3′
ANP	F: 5′‐CTCCGATAGATCTGCCCTCTTGAA‐3′
	R: 5′‐ GGTACCGGAAGCTGTTGCAGCCTA‐3′
BNP	5′‐AAGCTGCTGGAGCTGATAAGA‐3′
	5′‐GTTACAGCCAAACGACTGAC‐3′
KCNIP2	F: 5′‐GGCTGTATCACGAAGGAGGAA‐3′
	R: 5′‐CCGTCCTTGTTTCTGTCCATC‐3′
KCND2	F: 5′‐GCCGCAGCACCTAGTCGTT‐3′
	R: 5′‐CACCACGTCGATGATACTCATGA‐3′
SMAD4	F: 5′‐ACACCAACAAGTAACGATGCC‐3′
	R: 5′‐GCAAAGGTTTCACTTTCCCCA‐3′
β‐Actin	F: 5′‐TGGAATCCTGTGGCATCCATGAAAC‐3′
	R: 5′‐TAAAAACGCAGCTCAGTAACAGTCCG‐3′

*Note*: F represents forward primer and R represents reverse primer.

### 2.10 Western blotting (WB) assay

Protein samples from H9C2 cells were extracted with RIPA buffer (Thermo Fisher Scientific, USA) supplemented with protease and phosphatase inhibitors. The BCA kit (Thermo Fisher Scientific, USA) measured protein concentrations. SDS‐PAGE resolved proteins, which were then blotted onto PVDF sheets (Millipore, USA). These membranes were subsequently probed using primary antibodies targeting FLRT3, SMAD4, ANP, BNP, Bcl‐2, Bax, LC3I, LC3II, SCN5A, KCNIP2, KCND2 (all 1:1000, Abcam) and β‐actin (1:5000, Cell Signalling Technology) for normalization. Following primary incubation, blots were exposed to horseradish peroxidase‐linked secondary antibodies (1:5000, Abcam). Detection was accomplished via the ECL kit (Thermo Fisher Scientific) and visualized on a ChemiDoc platform (Bio‐Rad, USA).

### 2.11 Flow cytometry

For flow cytometric analysis, H9C2 cells were detached using trypsin–EDTA (Gibco, USA) and washed with phosphate‐buffered saline (PBS). To distinguish viable, apoptotic and necrotic cells, the cells were stained with Annexin V and propidium iodide (PI) according to the manufacturer's recommendations. To calculate the cell apoptosis rate, flow cytometry was carried out using a flow cytometer (BD Biosciences, USA), and the results were examined using FlowJo software (FlowJo LLC, USA).

### 2.12 Dual‐luciferase reporter assay

We used a dual‐luciferase reporter experiment to confirm the hypothesized SMAD4 binding sites on the FLRT3 promoter. H9C2 cells, at 80% confluence in 24‐well plates, were set up with either the wild‐type (WT: TTTCTAGA) or mutated (MUT: GGGTCAC) sequences of the FLRT3 promoter linked to the pGL3‐Basic vector (Promega, USA) ahead of the firefly luciferase gene. The pRL‐TK vector (Promega, USA) served as a control for normalization. Using Lipofectamine 2000 (Invitrogen, USA), cells were co‐transfected with the designed pGL3‐Basic vectors and the control pRL‐TK. Post 24‐h transfection, cells were processed to measure luciferase outputs with the Dual‐Luciferase Reporter Assay System (Promega, USA) as guided by the manufacturer. Firefly luciferase readings were adjusted based on Renilla luciferase. Tests were done in sets of three and replicated thrice.

### 2.13 Chromatin immunoprecipitation polymerase chain reaction (ChIP‐PCR)

ChIP‐PCR was used to assess SMAD4 binding to the FLRT3 promoter region. Chromatin immunoprecipitation assays were performed using H9C2 cells following the protocol provided by Abcam (Cambridge, MA, USA). Immunoprecipitation was carried out using an anti‐SMAD4 antibody, and a control IgG antibody was used in parallel to ensure the specificity of the SMAD4 binding. After immunoprecipitation, DNA was purified and PCR was performed using specific primers targeting the FLRT3 promoter region.

### 2.14 Statistical analysis

Each experiment was performed thrice, and results were shown as mean ± SD. The Student's t‐test was utilized for two‐group comparisons. For multiple group comparisons, we employed one‐way ANOVA followed by Tukey's test. Additionally, the discriminatory ability of the test was assessed using the AUC of the ROC curve. An AUC value close to 1.0 indicates an optimal diagnostic biomarker, while an AUC value of 0.5 indicates a lack of discriminatory power. A *p* < 0.05 indicated statistical significance. Analyses were done with SPSS 26.0 (IBM Corp.) and visualized using GraphPad Prism 8 (GraphPad Software, Inc.).

## 3 RESULTS

### 3.1 Hub module identification via WGCNA in SVT sequence data

**FIGURE 1:**
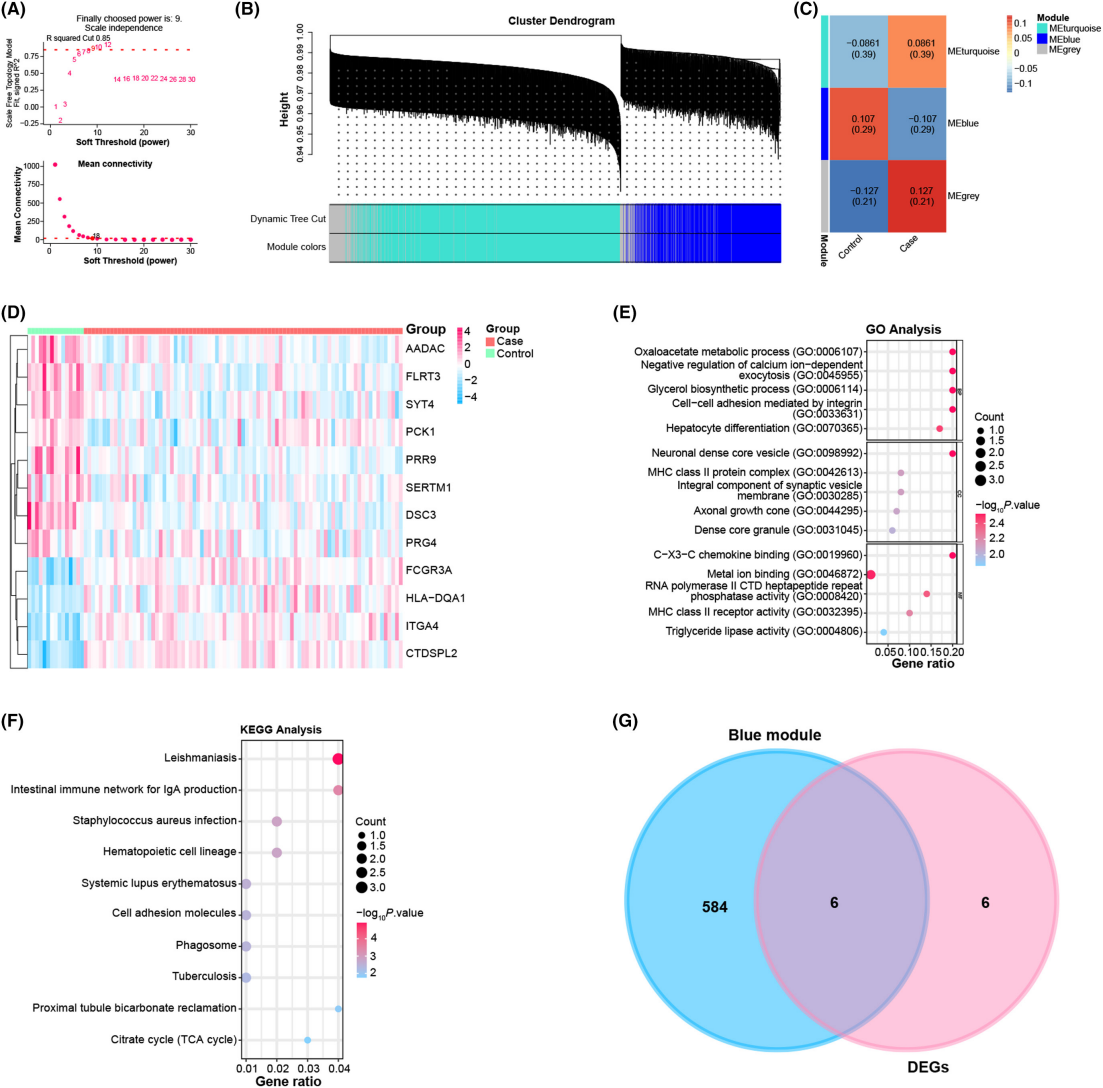
Co‐expression module analysis and identification of common genes. (A) Soft threshold power selection for WGCNA to achieve scale‐free topology. The X‐axis represents the soft threshold power (β) and the Y‐axis represents the scale‐free fit index. (B) Visualization of co‐expression modules identified by WGCNA. Each colour represents a different module containing co‐expressed genes. (C) Correlation between module signature genes and sample traits. The blue module shows a significant correlation with the sample traits, with a coefficient of 0.107 and −0.107. (D) Differential expression analysis of genes between case and control samples to identify upand downregulated DEGs. Red represents the case group and green represents the control group. (E, F) GO (E) and KEGG (F) pathway enrichment analysis revealed significantly enriched terms or pathways for DEGs. The X‐axis represents the Gene Ratio; the Y‐axis represents the GO Term or enriched pathway; the size of the dots represents the odds ratio; the colour of the dots represents the level of *p* value. (G) Venn diagram showing the overlap between DEGs and genes within blue co‐expression modules, with shared genes highlighted for further study.

From the results shown in Figure 1A, we successfully determined the optimal soft threshold power of 9 for fitting the scale‐free topology model. Subsequently, using the WGCNA method, we clustered the genes into different modules according to the co‐expression patterns between different samples, and each module was represented by a specific colour (Figure 1B). Among these, the blue module exhibited a significant correlation with the two groups of samples, with a coefficient of 0.107 (Figure 1C). This observation underscores the importance of the blue module in our study.

### 3.2 DEG profiling, pathway enrichment and intersection analysis in SVT samples

We screened 3190 upregulated DEGs and 3592 downregulated DEGs from 85 SVT samples and 15 health samples (Figure 1D). After identifying DEGs, we performed functional enrichment analysis. The results highlighted the significant enrichment of these DEGs in BP, CC and MF. Notably, GO terms such as triglyceride lipase activity, dense core particles and hepatocyte differentiation emerged as significant enrichments (Figure 1E). Likewise, KEGG pathways including cell adhesion molecules were also enriched (Figure 1F). Furthermore, among the top 10 DEGs and blue modules, we identified six overlapping genes, namely AADAC, DSC3, FLRT3, SYT4, PRR9 and SERTM1 (Figure 1G).

### 3.3 Expression analysis and ROC evaluation highlighting FLRT3 in SVT

**FIGURE 2:**
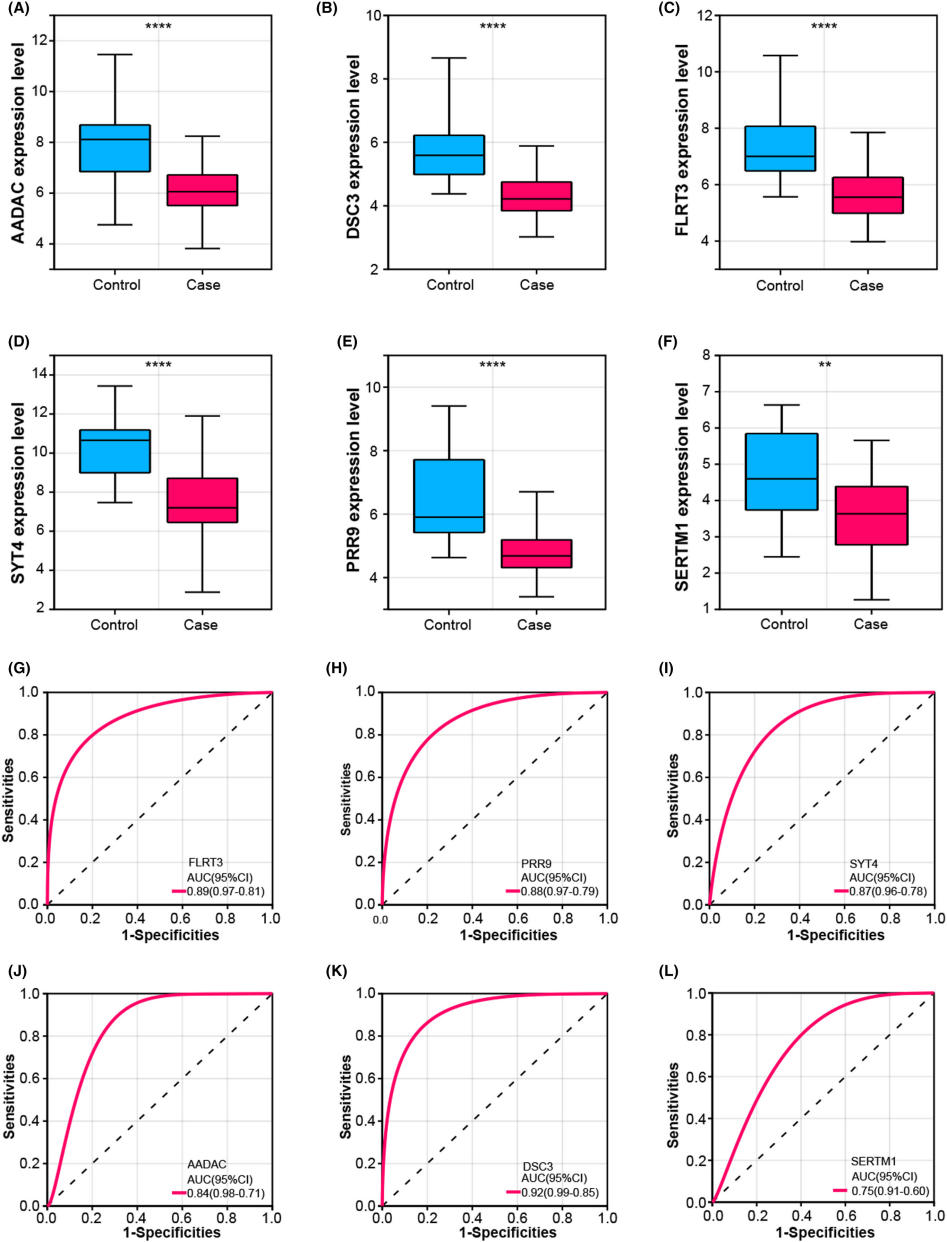
Expression analysis and ROC curves of overlapping genes. (A–F) Boxplots representing the expression patterns of six overlapping genes in the control and case groups. Blue represents the control group and red represents the case group. (G–L) ROC curve depicting the discriminatory performance of six overlapping genes in differentiating control and case samples. The AUC value is calculated to evaluate the accuracy of classification. The closer the AUC value is to 1, the better the performance of the model. **p < 0.01 and ****p < 0.0001.

We performed an in‐depth analysis of the expression patterns of the six overlapping genes in both the control and case groups. The findings revealed a pronounced upregulation of all six genes in the control group and downregulation in the case group (Figures 2A–F). To further evaluate the discriminative capability of these genes, we conducted a ROC curve analysis. In the analysed results, we observed that DSC3 had the highest AUC value of 0.92, closely followed by FLRT3 (AUC = 0.89), PRR9 (AUC = 0.88), SYT4 (AUC = 0.87), AADAC (AUC = 0.84) and SERTM1 (AUC = 0.75) (Figure 2G–L). Although DSC3 had the highest AUC value, it is of interest to note that axon guidance factors play a key role in the regulation of angiogenesis and vascular morphology. Whereas FLRT3, an axon guidance factor associated with neuronal cell development and shape, was prominent among these genes and had an AUC value of 0.89, we chose FLRT3 as a hub gene for further study given its importance in multiple biological processes. This selection was based on its critical role in the regulation of nerve cells and the vasculature. After the hub gene was confirmed by analysis, the expression of this gene was verified in the GSE79768 and GSE115574 data sets (Figure S1), and it was found that FLRT3 was generally significantly expressed low in the case groups of the two data sets.

### 3.4 Knockdown of FLRT3 inhibits cardiomyocyte apoptosis and promotes autophagy in H9C2 cells

**FIGURE 3:**
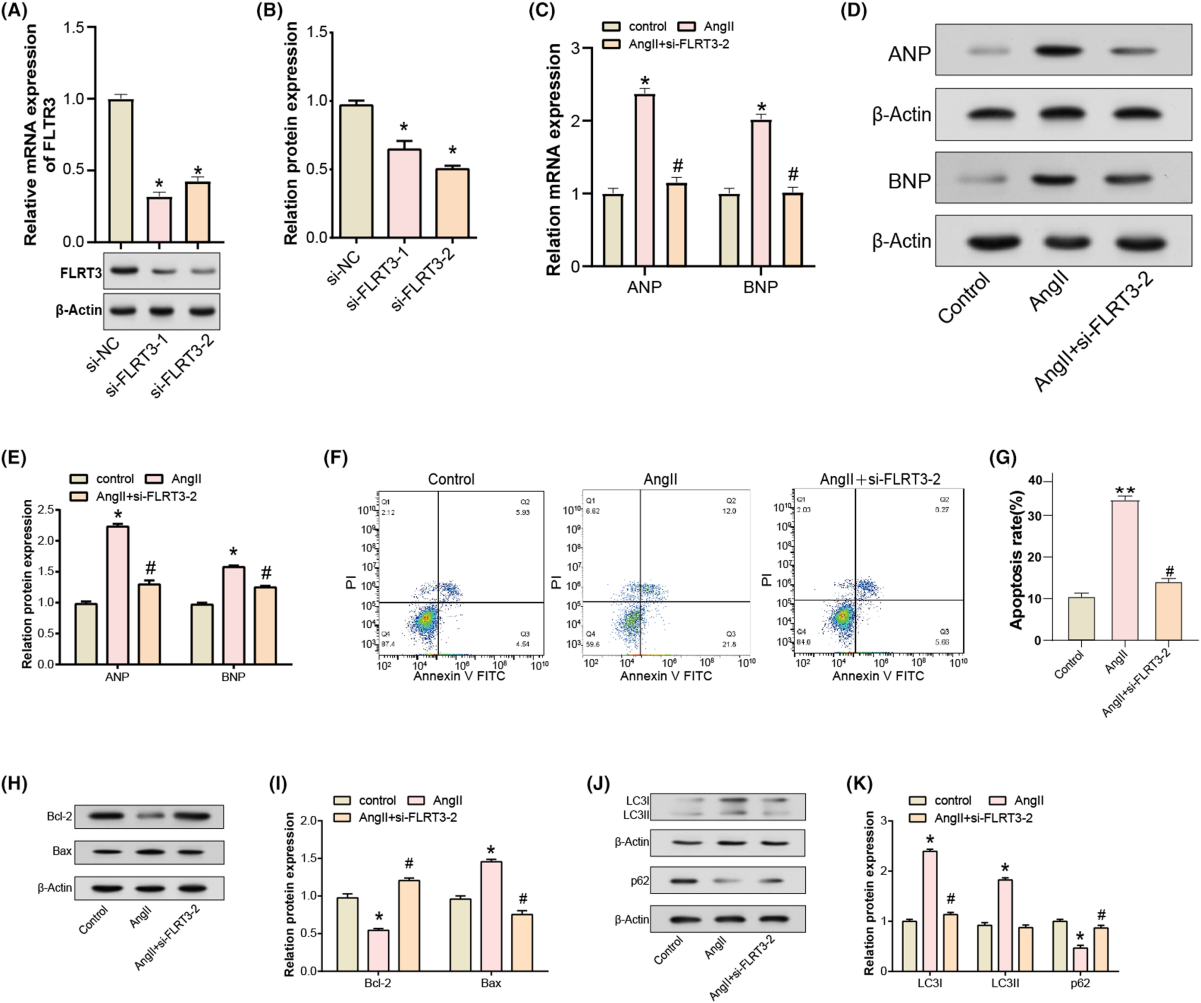
Knockdown of FLRT3 regulates cardiomyocyte apoptosis and autophagy in hypertrophic H9C2 cells. (A and B) qRT‐PCR and WB analysis of FLRT3 expression levels in H9C2 cells after siRNA‐mediated knockdown, with si‐FLRT3‐2 showing the most significant downregulation. (C–E) qRT‐PCR and WB analysis of ANP and BNP expression levels or protein levels in AngII‐induced H9C2 cells treated with or without si‐FLRT3‐2. (F and G) Flow cytometric analysis of H9C2 cell apoptosis following AngII stimulation, highlighting inhibition of apoptosis by si‐FLRT3‐2. (H and I) WB analysis of apoptosis‐related proteins, including Bcl‐2, Bax, c‐Caspase 3 and p53, illustrating their expression changes after AngII induction and si‐FLRT3‐2 co‐induction. (J and K) WB analysis of autophagy‐related proteins, including LC3I, LC3II, Beclin‐2 and p62, revealing the effects of Ang II and si‐FLRT3‐2 on the autophagy process. *p < 0.05 versus si‐NC group or control group, **p < 0.01 versus control group and ^#^ *p* < 0.05 versus Ang II group.

qRT‐PCR analysis showed that si‐FLRT3‐2 was the most downregulated after FLRT3 knockdown, and FLRT3 expression was significantly decreased in H9C2 cells, which was further confirmed by WB assay (Figure 3A,B). It is established that Ang II induction promotes hypertrophy in H9C2 cardiomyocytes. ANP is secreted in response to atrial hypertension or atrial dilation, while BNP, primarily released from ventricles, stands as a pivotal hormone associated with cardiac function.^21^ Both qRT‐PCR and WB analyses demonstrated that ANP and BNP expressions were significantly upregulated post‐Ang II stimulation. However, co‐induction with Ang II and si‐FLRT3‐2 attenuated the upregulation of ANP and BNP expressions (Figure 3C,D). Flow cytometry confirmed a significant increase in Ang II‐induced apoptosis. However, simultaneous introduction of si‐FLRT3‐2 inhibited the apoptotic phenomenon (Figure 3F,G). In addition, WB analysis showed apoptosis‐related proteins (Bcl‐2 and Bax) that Ang II induction resulted in decreased Bcl‐2 expression and increased Bax expression. In contrast, co‐induction with si‐FLRT3‐2 was reversible (Figure 3H,I). Moreover, WB analysis of autophagy‐related proteins (including LC3I, LC3II and p62) indicated that Ang II induction resulted in increased expression of LC3I and LC3II, and a decrease in p62 expression. In contrast, FLRT3 knockdown counteracted the effects (Figure 3J,K). These findings suggested that FLRT3 knockdown inhibited apoptosis and regulated autophagy in hypertrophic H9C2 cardiomyocytes.

### 3.5 Regulatory interactions between TGF‐β/SMAD4 signalling, FLRT3 and ion channel expression in H9C2 Cells

**FIGURE 4:**
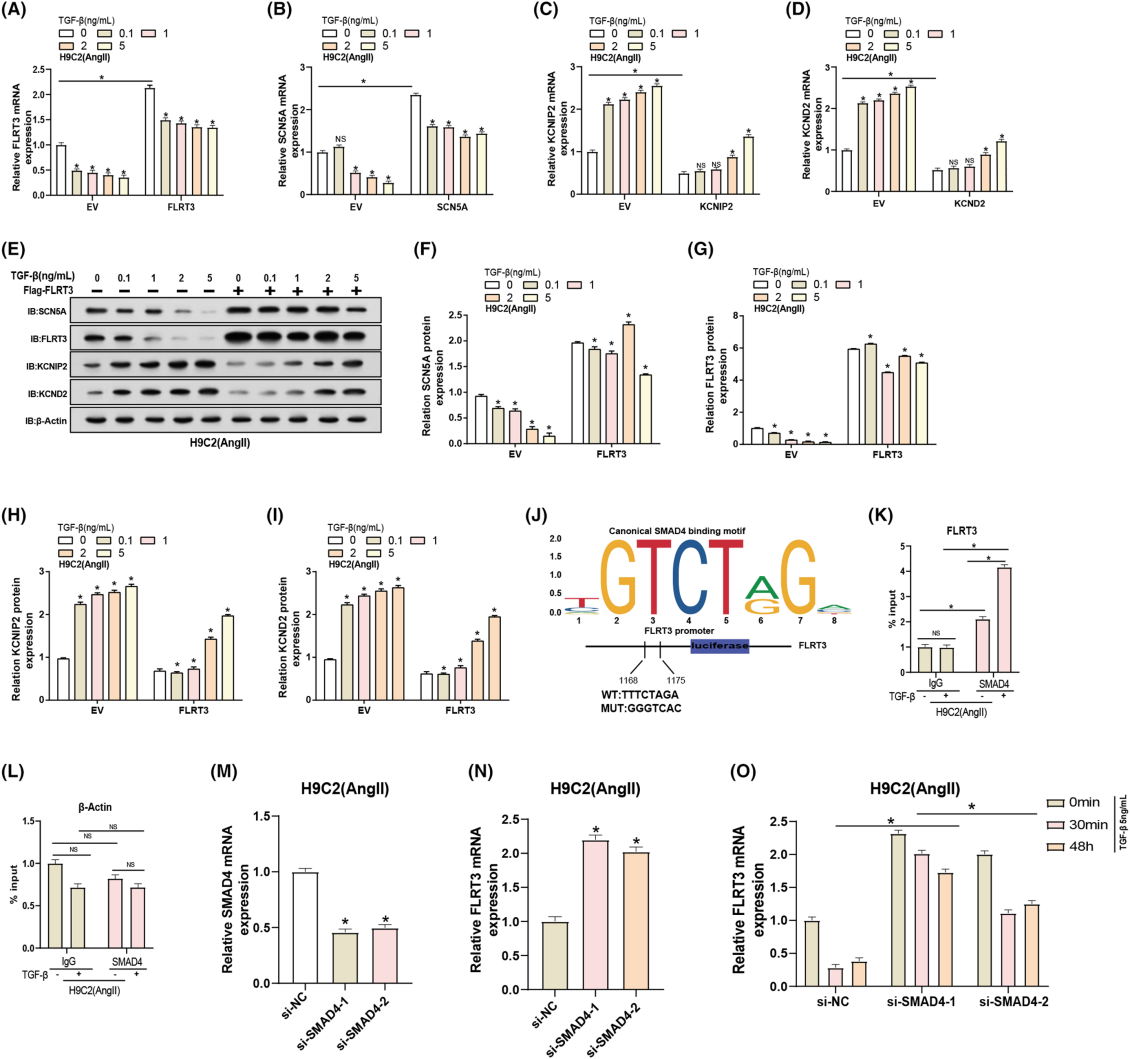
FLRT3 Regulates sodium and potassium channel protein expression via TGF‐β and SMAD4. (A–D) qRT‐PCR analysis depicting changes in FLRT3, SCN5A, KCNIP2 and KCND2 expression in H9C2 cells following TGF‐β treatment (0, 0.1, 1, 2 and 5 ng/mL). (E–I) WB analysis shows changes in protein expression of FLRT3, SCN5A, KCNIP2 and KCND2 after TGF‐β treatment. (J) Schematic diagram of predicting SMAD4 binding sites within the FLRT3 gene based on the JASPER database. Dual‐luciferase reporter assay demonstrates interaction between SMAD4 and FLRT3 promoter. (K and L) ChIP‐PCR analysis of the binding affinity of SMAD4 to the FLRT3 promoter in Ang II‐induced H9C2 cells treated with 5 ng/mL TGF‐β.β‐Actin served as a negative control. (M) qRT‐PCR analysis of SMAD4 expression changes after SMAD4 knockdown. (N) qRT‐PCR analysis of FLRT3 expression changes after SMAD4 knockdown. (O) qRT‐PCR analysis of FLRT3 expression in H9C2 cells (Ang II) treated with TGF‐β at different time points (0 min, 30 min and 48 h) after SMAD4 knockdown. *p < 0.05, ns means not significant. ‘+’ indicates cells treated with TGF‐β and ‘‐’ denotes untreated control cells. The empty vector (EV) is used as a control for comparison.

Utilizing qRT‐PCR, we assessed the mRNA expression levels of FLRT3, SCN5A, KCNIP2 and KCND2 in H9C2 cells treated with varying doses of TGF‐β (0, 0.1, 1, 2 and 5 ng/mL) while highlighting the empty vector (EV) as a control (Figure 4A–D). As the TGF‐β concentration increased, there was a dose‐dependent decline in FLRT3 expression, suggesting TGF‐β‐mediated suppression of FLRT3. A concomitant decrease was observed in SCN5A expression, reaching its nadir at 2 ng/mL of TGF‐β. Contrarily, both KCNIP2 and KCND2 exhibited parallel trends, with no significant expression alterations at 0.1 ng/mL and 1 ng/mL, but prominent upregulation at concentrations of 2 and 5 ng/mL. The trends discerned from WB analysis were in alignment with those obtained from qRT‐PCR (Figure 4F–I). Leveraging the JASPAR database, we identified the binding sites of FLRT3 and SMAD4. To validate these predictions, a dual‐luciferase reporter assay was conducted using vectors containing either the wild‐type (WT: TTTCTAGA) or mutated (MUT: GGGTCAC) FLRT3 promoter sequences. The luciferase activity indicated direct interactions between the predicted sites and SMAD4 (Figure 4J). ChIP‐PCR analysis confirmed the direct binding of SMAD4 to the FLRT3 promoter in the presence of 5 ng/mL TGF‐β, and this interaction was accentuated upon TGF‐β exposure (Figure 4K,L). Furthermore, qRT‐PCR analyses revealed that the expression of SMAD4 was significantly attenuated following transfection with two distinct siRNAs, leading to a concurrent upsurge in FLRT3 expression (Figure 4M,N). Subsequent TGF‐β treatment of H9C2 cells post‐SMAD4 knockdown across different durations (0 min, 30 min and 48 h) demonstrated a pronounced reduction in FLRT3 expression (Figure 4O). Collectively, these findings delineate a complex interplay between TGF‐β signalling and ion channel gene regulation, highlighting the critical role of SMAD4 in regulating FLRT3 expression in H9C2 cells.

## 4 DISCUSSION

The intricate aetiology and clinical manifestations of SVT necessitate a profound molecular understanding. In this context, WGCNA emerges as a robust tool, unravelling the complex landscape of gene–gene interactions.^22^ In our study, through WGCNA, we clustered genes into different modules based on co‐expression patterns, among which the correlation coefficient of the blue module with SVT was as high as 0.107, emphasizing its potential importance in the genetic architecture of SVT. After this, enrichment analysis of overlapping genes indicated key pathways and biological processes, especially those revolving around triglyceride lipase activity, dense core particles, leishmaniasis and *Staphylococcus aureus* infection, postulating their latent roles in the pathophysiology of SVT. Furthermore, Bonnet D et al. chronicled complications in neonates associated with atrioventricular valve hypoplasia, where SVT, staphylococcal endocarditis and persistent valvular disease were prominent.^23^ Such revelations underscore the multifaceted molecular dynamics of SVT and hint at external triggers and concomitant conditions modulating its onset. However, while these enriched results provide compelling insights, the precise nexus between them and SVT warrants deeper validation and exploration in subsequent studies.

In our comprehensive investigation into the molecular mechanisms of SVT, identifying and validating pivotal molecules is imperative. Through rigorous overlap analysis, FLRT3 emerged as a potential diagnostic biomarker. A thorough examination of the expression profiles of the six intersecting genes revealed a marked downregulation of FLRT3 in SVT samples relative to controls. This distinct expression differential underscores FLRT3's potential role in cardiac rhythm modulation or perhaps its involvement in thwarting arrhythmogenesis. FLRT3, denoted as Fibronectin Leucine Rich Transmembrane Protein 3, is a member of the FLRT family and has traditionally been associated with processes like neuronal development and axon guidance.^24^, ^25^ Within this realm, it functions as a cell adhesion molecule, facilitating neuronal connectivity.^26^ While its prominence in neural contexts is recognized, contemporary studies have broadened its significance beyond the neural landscape.^27^, ^28^ Emerging evidence elucidates its involvement in diverse cellular processes encompassing tissue development, immune modulation and even cancer progression.^29^, ^30^ Intriguingly, before our study, the role of FLRT3 in cardiac physiology and, specifically, within the backdrop of SVT, remained relatively uncharted.

Ang II is a potent mediator of multiple physiological processes, including blood pressure regulation and fluid balance.^31^ Many studies have utilized Ang II‐induced H9C2 cell hypertrophy as a reliable model to simulate cardiac hypertrophic conditions in vivo, allowing researchers to elucidate potential molecular mechanisms and therapeutic targets.^32^, ^33^ Based on this, we selected Ang II‐induced H9C2 cardiomyocytes for study. In our results, FLRT3 expression was significantly decreased in H9C2 cells after silencing, suggesting its potential role in regulating cellular processes during cardiac hypertrophy. Furthermore, ANP and BNP expression were significantly upregulated after Ang II induction, but the knockdown of FLRT3 counteracted this effect. ANP and BNP are typically released in response to conditions such as atrial dilation or ventricular dysfunction, respectively, and are key markers of heart health and function.^34^, ^35^ The secretion of BNP is usually associated with ventricular dilation and increased myocardial load.^36^ For example, Cheng M L et al highlighted the prognostic value of BNP in predicting prognosis in patients with heart failure.^37^ The literature by Nishikimi T et al also highlights BNP as a primary target in patients with AF.^38^ Furthermore, elevated levels of these natriuretic peptides have been associated with adverse cardiac events in numerous studies.^39^, ^40^ Therefore, understanding the complex balance and regulatory mechanisms is crucial to advance therapeutic strategies for SVT.

Autophagy and apoptosis, both essential cellular processes, play instrumental roles in cardiovascular diseases, including cardiac hypertrophy.^41^ While autophagy involves the degradation and recycling of cellular components to maintain cellular homeostasis, apoptosis, the process of programmed cell death, ensures the elimination of damaged or unnecessary cells.^42^ Disturbances in the balance between these two processes can lead to pathological conditions, including cardiac hypertrophy and arrhythmias.^43^, ^44^ Dysregulated autophagy and apoptosis can induce myocardial cellular anomalies, culminating in cell death and subsequent fibrotic remodelling, thereby predisposing the myocardium to arrhythmic events.^45^ The modulation of autophagic activity has the potential to suppress cardiac hypertrophy, which in turn can modulate manifestations of arrhythmias.^43^ It has been confirmed that FLRT3 is involved in the regulation of the apoptosis pathway. For instance, Yang M et al. reported that ectopic overexpression of FLRT3 could inhibit epithelial–mesenchymal transition (EMT) in colorectal cancer and promote cell apoptosis.^11^ In this context, we hypothesize that FLRT3 may be involved in these processes. Our flow cytometry analyses revealed a pronounced inhibition of Ang II‐induced apoptosis upon the introduction of si‐FLRT3‐2. WB further validated these observations, indicating a shift in the balance of apoptotic‐related proteins in favour of cell survival, as evidenced by increased Bcl‐2 and reduced Bax expressions. Furthermore, perturbations in autophagy‐related proteins, such as LC3, have been linked to the progression of cardiac hypertrophy, as highlighted in the work by Nakai A et al.^46^ In our investigations, Ang II induction significantly altered the expression levels of autophagy markers, LC3I and LC3II, while decreasing p62 expression. However, with the knockdown of FLRT3, these effects were counteracted, signifying the crucial role of FLRT3 in modulating autophagy in hypertrophic cardiomyocytes. Collectively, our findings illuminate the intricate roles of FLRT3 in cardiomyocyte apoptosis and autophagy, providing insights into prospective therapeutic strategies for cardiac hypertrophy and associated pathologies.

The TGF‐β signalling pathway, primarily mediated by SMAD proteins, controls various cellular activities such as growth, differentiation and apoptosis.^47^, ^48^ It is increasingly apparent that ion channel gene expression can be modulated by growth factors and cytokines, underscoring the integration of electrophysiological and cellular signalling pathways.^49^, ^50^ Our results suggested a multifaceted interaction between TGF‐β signalling and ion channel gene regulation, particularly FLRT3 and SMAD4. FLRT3 expression decreased in a dose‐dependent manner with increasing TGF‐β concentration, suggesting a potential inhibitory effect. This coincides with a decrease in SCN5A expression. However, the ion channel‐related genes KCNIP2 and KCND2 showed a different response, being upregulated at higher TGF‐β concentrations. This suggests that TGF‐β may regulate ion channel expression in H9C2 cells through SMAD4, a complex regulatory mechanism. chIP‐PCR analysis confirmed a direct interaction between SMAD4 and the FLRT3 promoter, which was exacerbated by TGF‐β exposure. A study by Kaur K et al. highlighted that TGF‐β1 can influence sodium and potassium channel gene expression, impacting cellular electrophysiology, particularly in cardiac tissues.^51^ Based on our empirical data and observations, it is plausible to suggest that FLRT3 may play a role within this dynamic system, potentially acting as an intermediary or regulator of ion channel activity through TGF‐β/SMAD4 signalling.

## 5 CONCLUSION

**FIGURE 5:**
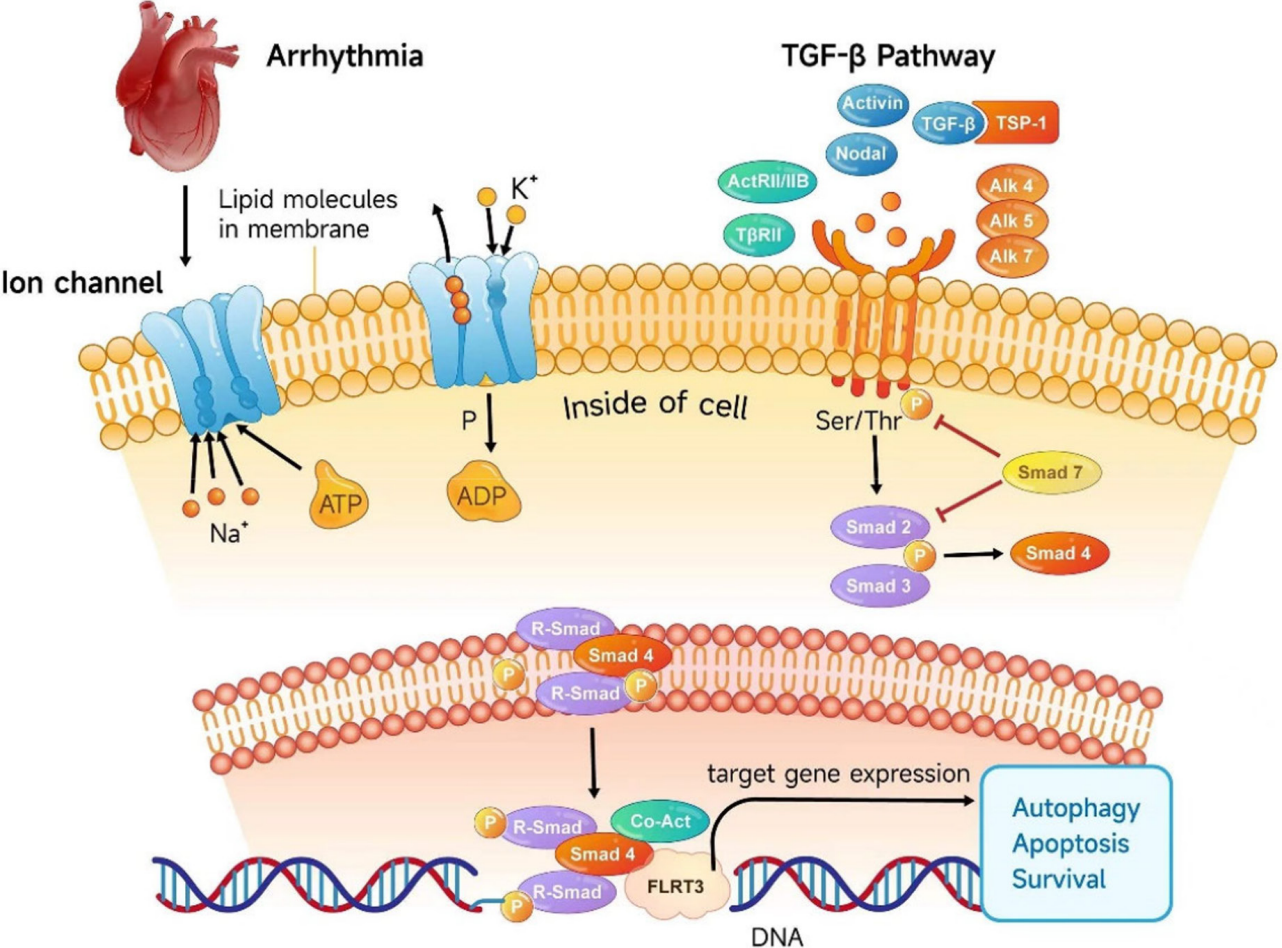
Mechanistic illustration of FLRT3 as a therapeutic target for SVT through the regulation of the ion channel remodelling and TGF‐β/SMAD4 signalling pathway.

Our integrative research unveiled the intricate interactions among TGF‐β signalling, SMAD4 and FLRT3. Experimental outcomes indicated that knockdown of FLRT3 provides a protective effect against cardiomyocyte apoptosis and orchestrates autophagy in hypertrophic cardiomyocytes. Such observations underscore the potential of FLRT3 as a therapeutic target for SVT. Furthermore, our study elucidated the modulatory roles of the TGF‐β/SMAD4 signalling pathway in the FLRT3‐mediated regulation of sodium and potassium ion channel (Figure 5). Insights into this mechanism offer a more comprehensive understanding of ion channel remodelling in the context of SVT.

## 6 LIMITATIONS

Despite the robustness of our findings, this study has inherent limitations. Firstly, our research largely hinges on in vitro experiments involving H9C2 cardiomyocytes, which, although a widely accepted model, may not capture the full complexity of in vivo human cardiac tissues. Secondly, while our findings point towards a regulatory relationship among TGF‐β, SMAD4 and FLRT3, establishing causality warrants more extensive investigations. There are some limitations in the methodology (e.g. regression) and experimental preprocessing of this study that have not yet been realized. Lastly, the potential feedback loops and other intermediary factors modulating ion channel expression remain unexplored, necessitating a more comprehensive understanding of the regulatory network.

## AUTHOR CONTRIBUTIONS

**Yang Pang:** Conceptualization (equal); software (equal). **Ye Xu:** Formal analysis (equal); visualization (equal). **Qingxing Chen:** Investigation (equal); writing – original draft (equal). **Kuan Cheng:** Formal analysis (equal); software (equal). **Yunlong Ling:** Data curation (equal); visualization (equal). **Jun Jang:** Investigation (equal); writing – review and editing (equal). **Junbo Ge:** Methodology (equal); visualization (equal). **Wenqing Zhu:** Data curation (equal); supervision (equal).

## FUNDING INFORMATION

This study was supported by Shanghai Science and Technology Innovation Fund (No. 17DZ1930100), Shanghai Municipal Commission of Economy and Informatization (No. GYQJ‐2018‐2‐05), Project of Shanghai Science and Technology Committee (No. 21S31906902) and Shanghai Municipal Key Clinical Specialty (No. shslczdzk01701).

## CONFLICT OF INTEREST STATEMENT

All authors declare that there are no conflicts of interest.

## INFORMED CONSENT STATEMENT

All participants gave written informed consent before participation and sample collection. The consent process was designed to ensure that participants understood the objectives, potential benefits, risks and procedures associated with the study, as well as their rights to privacy and their ability to withdraw from the study at any point. Procedures adhered to the 1964 Helsinki Declaration and its later amendments. This study was approved by the ethics committee of Zhongshan Hospital, Fudan University (Approval No. B2018‐065).

## Supporting information


Data S1.

Table S1.

Figure S1.

## Data Availability

The data sets used and/or analysed during this study are available from the corresponding author on reasonable request.
