# Supplementary figures and images for: FLRT3 and TGF‐β/SMAD4 signalling: Impacts on apoptosis, autophagy and ion channels in supraventricular tachycardia
Source: J Cell Mol Med. 2024 Mar 20;28(7):e18237. doi: 10.1111/jcmm.18237 (PMC10955158; doi:10.1111/jcmm.18237)

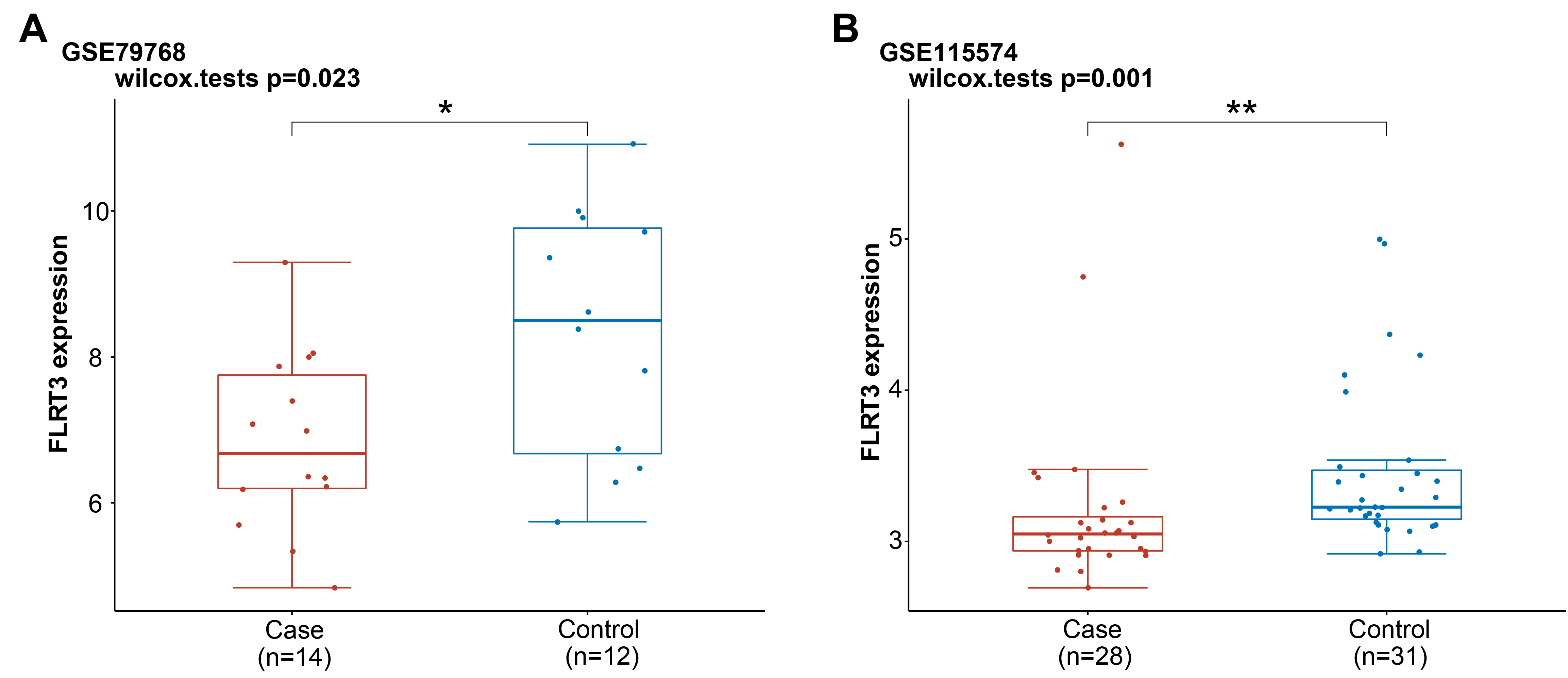

Supplement: Supplementary file 3 — Figure S1. [file JCMM-28-e18237-s002.tif]
