# Supplementary material for: FLRT3 and TGF‐β/SMAD4 signalling: Impacts on apoptosis, autophagy and ion channels in supraventricular tachycardia
Source: J Cell Mol Med. 2024 Mar 20;28(7):e18237. doi: 10.1111/jcmm.18237 (PMC10955158; doi:10.1111/jcmm.18237)
Supplement: Supplementary file 1 — Data S1. [file JCMM-28-e18237-s003.docx]

Dear editor,

Thank you for your advice. The required caption were listed as bellows:

**Supplementary Figure 1 caption：**the expression levels of FLRT3 in two distinct studies. A depicts data from study GSE79768, highlighting a statistically significant difference in FLRT3 expression between cases (n=14) and controls (n=12) with a p-value of 0.023. B represents study GSE115574, showing a more pronounced difference in expression levels between cases (n=28) and controls (n=31) with a p-value of 0.001. Both parts underline the potential role of FLRT3 in the studied condition, with statistical annotations indicating significance levels.

**Supplementary data table caption**：Table provides detailed demographic and clinical information of patients from our study. It is divided into two main sections:The first section lists the patient number (Num), gender, age, and other diseases for control group（15）. This part starts with patient Num 1, a 52-year-old male with no other listed diseases, and continues similarly for other male patients；The second section details the same categories for case group（85）, including patient number, gender, age, diagnosis, medical history in years (Medical History (Y)), onset age, and other diseases.
